# Pharmacological Investigation of the Wound Healing Activity of *Cestrum nocturnum* (L.) Ointment in Wistar Albino Rats

**DOI:** 10.1155/2016/9249040

**Published:** 2016-02-25

**Authors:** Hemant Kumar Nagar, Amit Kumar Srivastava, Rajnish Srivastava, Madan Lal Kurmi, Harinarayan Singh Chandel, Mahendra Singh Ranawat

**Affiliations:** ^1^Department of Pharmacology, Truba Institute of Pharmacy, Karond Gandhi Nagar, Bhopal, Madhya Pradesh 462038, India; ^2^Department of Pharmacology, Sapience Bioanalytical Research Lab, Bhopal, Madhya Pradesh 462021, India; ^3^Faculty of Pharmacy, Moradabad Educational Trust Group of Institutions, Moradabad, Uttar Pradesh 244001, India; ^4^Bhupal Nobles' College of Pharmacy, Udaipur, Rajasthan 313002, India

## Abstract

*Objectives.* The present study was aimed at investigating the wound healing effect of ethanolic extract of* Cestrum nocturnum* (L.) leaves (EECN) using excision and incision wound model.* Methods.* Wistar albino rats were divided into five groups each consisting of six animals; group I (left untreated) considered as control, group II (ointment base treated) considered as negative control, group III treated with 5% (w/w) povidone iodine ointment (Intadine USP), which served as standard, group IV treated with EECN 2% (w/w) ointment, and group V treated with EECN 5% (w/w) ointment were considered as test groups. All the treatments were given once daily. The wound healing effect was assessed by percentage wound contraction, epithelialization period, and histoarchitecture studies in excision wound model while breaking strength and hydroxyproline content in the incision wound model.* Result*. Different concentration of EECN (2% and 5% w/w) ointment promoted the wound healing activity significantly in both the models studied. The high rate of wound contraction (*P* < 0.001), decrease in the period for epithelialization (*P* < 0.01), high skin breaking strength (*P* < 0.001), and elevated hydroxyproline content were observed in animal treated with EECN ointments when compared to the control and negative control group of animals. Histopathological studies of the EECN ointments treated groups also revealed the effectiveness in improved wound healing.* Conclusions. *Ethanolic extract of* Cestrum nocturnum *(EECN) leaves possesses a concentration dependent wound healing effect.

## 1. Introduction

Wound is defined as the disruption of the anatomic and cellular continuity of tissue caused by chemical, physical, thermal, microbial, or immunological injury to the tissue. Wound healing processes consist of integrated cellular and biochemical cascades leading to reestablishment of structural and functional integrity of the damaged tissue [1]. Various growth factors such as transforming growth factor beta (TGF-*β*), platelet activation factor (PAF), epidermal growth factor (EGF), and platelet-derived growth factors (PDGF) seem to be necessary for the initiation and promotion of wound healing [2].

Various treatment options (analgesics, antibiotics, and nonsteroidal anti-inflammatory drugs) are available for the wound management but majority of these therapies produce numerous unwanted side effects [3, 4]. In recent years, several studies have been carried out on herbal drugs to explicate their potential in wound management and these natural remedies proved their effectiveness as an alternative treatment to available synthetic drugs for the treatment of wound [5]. Many natural herbs have been pharmacologically reported possessing potent wound healing activity [6].

Solanaceae (*Cestrum nocturnum *(L.) family) is an evergreen woody shrub growing to 4 m in height. Leaves are dark green, oblong-ovate to oblong-lanceolate in shape with a pointed tip and 6–20 cm long with an entire margin. The nocturnal flowers are greenish white, having powerful sweet intoxicating fragrance [7]. Recent studies demonstrated the presence of important bioactive phytoconstituents in* Cestrum nocturnum *like alkaloids, flavonoids, glycosides, steroids, phenols, and essential oils [8]. It was also found to possess anti-inflammatory, antimicrobial, local anesthetic, and antioxidant properties that rationalized its more supportive and significant role as ideal wound healing drug [9–11].

Although need for scientific validation of herbal plants of ethnopharmacological relevance before these could be recommended for wound recovery as drugs is essential, therefore in the light of abovementioned facts about the plant, present study was designed to evaluate the wound healing potential of* Cestrum nocturnum *(L.) using excision and incision wound model in Wistar albino rats.

## 2. Materials and Methods

### 2.1. Plant Collection and Authentication

Fresh leaves of* Cestrum nocturnum *were collected in the month of February, locally from Govindpura, Bhopal, Madhya Pradesh, India. Plant material was identified and authenticated by Dr. Zia-Ul Hasan, Head of Department, Department of Botany, Safia Science College, Bhopal, and a specimen voucher (500/Bot/Safia/14), deposited in the Department of Pharmacology, Sapience Bioanalytical Research Lab, Bhopal, for future reference.

### 2.2. Extraction

The leaves of* Cestrum nocturnum *were shade-dried for 2 weeks, then pulverized to a coarse powder, and passed through sieve number 20 to maintain uniformity. Coarsely dried powder of the leaves was first defatted with petroleum ether (60–80°C) for 72 h to remove fatty materials and then extracted with ethanol (60–70°C) using soxhlet apparatus for 36 h; the extract was collected, filtered through Whatman filter paper, and concentrated in vacuum under reduced pressure and the dried extract was stored at 4°C for further study. The percentage yield of the extract was calculated.

### 2.3. Preliminary Phytochemical Screening

Ethanolic extract of* Cestrum nocturnum* leaves (EECN) was subjected to various phytochemical screening tests for the identification of the phytoconstituents present in* Cestrum nocturnum* leaves using standard procedures [12].

### 2.4. Determination of Total Polyphenolic and Total Flavonoid Contents

The total polyphenols content of the EECN was measured by UV spectrophotometrically according to the Folin-Ciocalteu method using gallic acid as a standard [13]. 0.1 mL of the extract solution was mixed with 0.5 mL of Folin-Ciocalteu reagent in a test tube and volume was made up to the 3 mL with distilled water. After 3 min of incubation, 2 mL of 20% sodium carbonate (Na_2_CO_3_) solution was added and mixed thoroughly. The resulting mixture was incubated for 5 min at 50°C and cooled at room temperature. Absorbance of the mixture was measured at 650 nm against the reagent blank. All measurements were carried out in triplicate. Content of phenolic compounds was expressed as mg of gallic acid equivalents (GAE)/g of dry extract using the linear equation obtained from calibration curve of the standard gallic acid graph. The coefficient of determination (*R* ^2^) was 0.9971.

The total flavonoid content of the EECN was determined according to aluminum chloride method using quercetin as standard [14]. A volume of 0.5 mL of aluminum chloride (AlCl_3_) ethanol solution (2%) was added to 0.5 mL of sample solution. Extract sample was evaluated at a final concentration of 0.1 mg/mL. After 1 h of incubation at room temperature, the absorbance was measured at 420 nm. All measurements were carried out in triplicate. The total flavonoid content was calculated as mg of quercetin equivalents (QE)/g of dry extract using the linear equation obtained from calibration curve of the standard quercetin graph. The coefficient of determination (*R* ^2^) was 0.9964.

### 2.5. Animals

Healthy Wistar albino rats weighing between 180 and 200 g were used for the present study. Animals were procured from the authorized animal house of Sapience Bioanalytical Research Lab, Bhopal, Madhya Pradesh. The animals were acclimatized to the standard laboratory conditions in cross ventilated animal house at 25 ± 2°C, relative humidity 44–56%, and light and dark cycle of 12 : 12 hours and fed with standard diet and water* ad libitum *during the study. The study protocol was approved by the Institutional Animal Ethics Committee (Approval Number 1413/PO/a/11/CPCSEA) as per Committee for the Purpose of Control and Supervision of Experiments on Animals guidelines, India.

### 2.6. Preparation of Formulation

Two types of ointment formulations, 2% and 5% (w/w), were prepared from the extract where 5 and 10 g of the extract were incorporated into 100 g of simple ointment base British Pharmacopoeia (BP), respectively [15]. Povidone iodine ointment (5% w/w) was used as a standard drug for comparing the wound healing potential of the extract.

### 2.7. Wound Healing Activities

For excision and incision wound model, animals were divided into five groups each consisting of six animals as follows: group I, left untreated and considered as control, group II, which served as negative control (ointment base treated), group III, which served as standard and was treated with 5% (w/w) povidone iodine ointment USP (Intadine), groups IV and V which were treated with 2% and 5% (w/w) ointments of extract, respectively. All the treatments were given once daily.

### 2.8. Excision Wound Model

Excision wound was created as per the method described [16]; five groups of animals each containing six rats were shaved on the dorsum portion using depilatory cream (Reckitt Benckiser, Inc., UK) and anesthetized using ketamine hydrochloride (50 mg/kg, i.p., body weight). An impression was made on shaved dorsal region and area of the wound to be created was marked. A full thickness excision wound with a circular area of 314 mm^2^ was created along the marking using toothed forceps, a surgical blade, and pointed scissors. Rats were left undressed to the open environment. The simple ointment base, formulated extract ointment, and standard drug were applied once daily from the day of the operation until the complete healing. In this model, wound contraction and epithelialization period were evaluated. Wound contraction was measured as percent contraction every 4th day after wound formation. At the end of the study, all the rats were anesthetized and from the healed wounds, specimen samples of tissue were collected from each rat, leaving a 5 mm margin of normal skin around the edges of the healed wound. Specimen tissues were stored in 10% formalin solution and used for histopathological and biochemical studies.

### 2.9. Incision Wound Model

Incision wound was created according to the method already described [17]. The animals were grouped and treated the same as in the excision wound model. All rats were anesthetized using ketamine hydrochloride (50 mg/kg, i.p., body weight). Paravertebral incision of 6 cm length was made through the entire thickness of the shaved skin, on either side of the vertebral column of the rats with the help of a sharp scalpel. After complete hemostasis, the wound was stitched by means of interrupted sutures placed approximately 1 cm apart using black silk surgical thread (number 000) and a curved needle (number 11). After stitching, the wound was left undressed and animals were treated daily for 10 days. On the 10th day, all rats were anesthetized and sutures were removed and tensile strength of cured wound skin was measured using tensiometer.

### 2.10. Wound Healing Evaluation Parameters

#### 2.10.1. Measurement of Wound Contraction and Epithelialization Period

In the excision wound model, wound area was measured by tracing the wound with the help of transparent sheet using millimeter based graph paper on days 0, 4, 8, 12, and 16 for all groups. Wound contraction was measured every 4th day until complete wound healing and represented as percentage of healing wound area [18]. Percentage of wound contraction was calculated taking the initial size of the wound as 100% using the following formula:

(1) % wound contraction=Intial wound area−Specific day wound areaIntial wound area×100.

Epithelialization period was calculated as the number of days required for falling off the dead tissue remnants of the wound without any residual raw wound [19].

### 2.11. Measurement of Tensile Strength

The tensile strength of a healing skin wound indicates the degree of wound healing. It represents how much the healed tissue resists to breaking under tension and may identify the quality of healing tissue. On the 10th day, all the animals were anesthetized by injecting ketamine hydrochloride (50 mg/kg, i.p., body weight), the sutures were removed, and the healed tissue was excised from all animals. Tensile strength of excised tissue was measured with the help of tensiometer [20].

### 2.12. Hydroxyproline Estimation

Excised wound tissues from all rats were analyzed for the estimation of hydroxyproline. Tissues were dried in a hot air oven at 60°C to constant weight and were hydrolyzed in 6 N HCl for 4 h at 130°C. The hydrolysates were then neutralized to pH 7.0 and were subjected to Chloramine-T oxidation for 20 min. After 5 min, the reaction was terminated by the addition of 0.4 M perchloric acid and developed color with Ehrlich reagent at 60°C. After thorough stirring the samples were analyzed at 557 nm in ultraviolet (Systronics-2203) spectrophotometer. The hydroxyproline content in the tissue samples was calculated using a standard curve of the pure L-hydroxyproline [21].

### 2.13. Histopathological Study

At the end of the study, all the animals were anesthetized using ketamine and specimens of wound tissue were collected and preserved in glass vials containing 10% formalin solution for histological examination. Sections of wound tissue specimens (about 5 *μ*m thickness) were prepared by microtomy and stained with hematoxylin and eosin (H&E) dye for histological examination.

### 2.14. Statistical Analysis

The results are expressed as mean ± standard error of mean (SEM). The statistical significance was analyzed using one-way analysis of variance (ANOVA) followed by Tukey-Kramer Multiple Comparisons Test employing statistical software, GraphPad, InStat 3. Differences between groups were considered significant at *P* < 0.05 levels.

## 3. Results

### 3.1. Preliminary Phytochemical Screening

The percentage yield of EECN was found to be 11.78% w/w. The preliminary phytochemical investigation of the EECN revealed the presence of alkaloids, flavonoids, glycosides, tannins, triterpenoids, polyphenols, carbohydrates, and proteins (Table 1).

### 3.2. Determination of Total Polyphenolic and Total Flavonoid Contents

The phenolic and flavonoid contents of the EECN are represented in Table 1. The amount of phenolic contents was 238.64 ± 1.29 mg of gallic acid equivalents (GAE)/g of extract. The flavonoid content was 61.39 ± 0.57 mg of quercetin equivalents (QE)/g of extract.

### 3.3. Effect of EECN on Percentage Wound Contraction and Epithelialization Period

**Figure 1:**
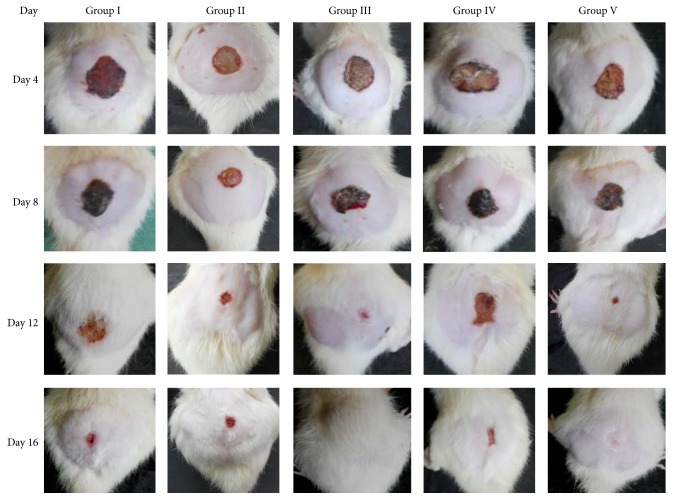
Photographs of wound repair at different time interval in excision wound model in rats.

During the course of treatment the extract was found to show its preliminary effect from day 4 up to day 16 (Figure 1). The credentials found on day 16 anonymously favor the potential curative effect of the test drug (Table 2), which shows its maximum significant effect by increasing wound contraction with respect to control (*P* < 0.001) and ointment base treated (*P* < 0.001) and standard groups (*P* < 0.01) that proportionally confer healing process. As per rate of epithelialization concern the test drug was found to show its contributory role in the accelerating epithelialization rate and required lesser time to complete epithelialization process (*P* < 0.01) as compared to control and the ointment base treated group (Table 2).

### 3.4. Effect of EECN on Tissue Hydroxyproline Content

Increased hydroxyproline content ultimately responsible for increasing the collagen level confirmed the increased viability or microcirculation of collagen fibrils around the wound area. The hydroxyproline level was found to be significantly elevated (*P* < 0.01) in treated group animals in a concentration dependent manner in comparison to control and ointment base treated group (Table 3). The relative order for different groups in accordance to collagen stability or wound strength was at standard 5% povidone iodine > extract 5% > extract 2% > ointment base treated > control.

### 3.5. Effect of EECN on Tensile Strength of the Wound

An ideal wound healing agent must have the property of increasing the viability of collagen fibrils around the wound area that increases the tensile strength of the wound that was assessed by evaluating the tensile strength of the healed wound using tensiometer (Table 4). The EECN was found to possess significant concentration dependent action in increasing the tensile strength as compared to control and ointment base treated group (*P* < 0.01 and *P* < 0.001).

### 3.6. Histopathological Study

**Figure 2:**
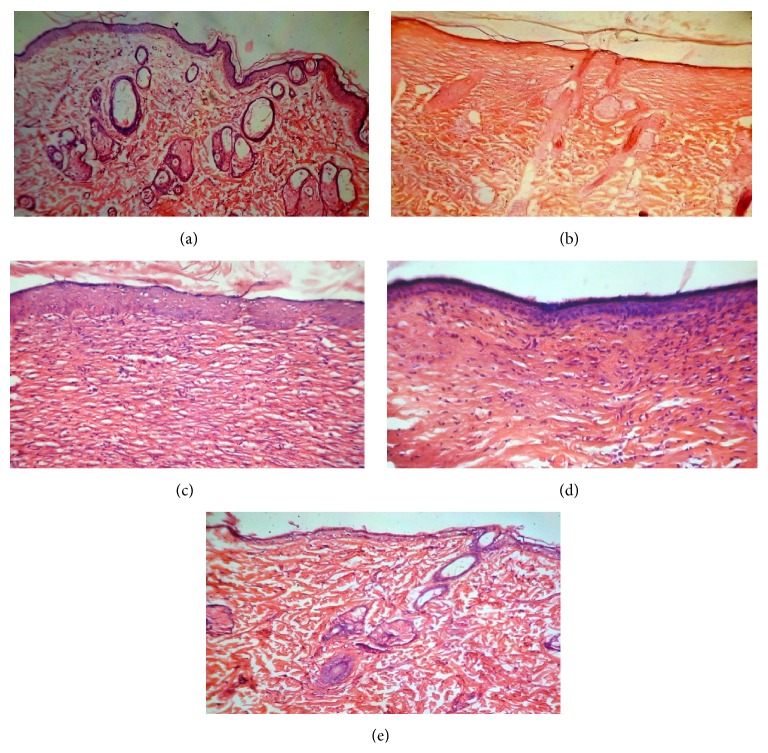
Photomicrograph of histopathological section of wound tissue of rats (stained with H&E, 40x magnification). (a) Histopathological section of group I (control) animal wound tissue. (b) Histopathological section of group II (ointment base treated) animal wound tissue. (c) Histopathological section of group III (standard) animal wound tissue. (d) Histopathological section of group IV (2% w/w ointment) animal wound tissue. (e) Histopathological section of group V (5% w/w ointment) animal wound tissue.

The histopathological studies of the tissue of the excision wound were performed on the 16th day and histopathological features of the tissue of all groups of animals are shown in Figures 2(a)–2(e). Section of group I (control) animals showed inflammatory cells, reduced collagen fibers, fibroblast cells, and blood vessels; there is also a presence of visible scar tissue (Figure 2(a)). Group II (ointment base treated) displayed the necrotic cells and less collagen fibers and blood vessels (Figure 2(b)). Group III (standard) showed complete tissue regeneration which was evident by increased fibroblast cell, collagen fibers, and blood vessels and reduced inflammatory cells (Figure 2(c)). Section of group IV (2% w/w ointment) showed less cellular necrosis along with increased collagen fibers and blood vessels (Figure 2(d)). However group V (5% w/w ointment) showed prominently increased fibroblast cells, blood vessels, and well organized collagen fibers as compared to the control (Figure 2(e)). Extract ointment treated and standard groups also showed the proliferation of epithelial tissue along with keratinization.

## 4. Discussion

Wound healing is an intricate process following damage to the skin and other soft tissues of the body. Wound healing involves the dynamic process of multiple biochemical consequences towards restoration of the damaged cellular structure to its regular and original state [22]. A classical cascade of wound healing involves three sequential and overlapping phases: inflammation, proliferation, and remodeling [23]. Topical application of prepared ointments (2% and 5% w/w) of EECN improved the wound healing in both excision and incision wound model in rats.

The preliminary qualitative phytochemical screening of the EECN showed the presence of alkaloids flavonoids, terpenoids, glycosides, and tannins. Quantitative analysis of the EECN revealed rich amount of phenolic and flavonoidal content in the leaves of* Cestrum nocturnum*. Recent studies suggested the valuable role of flavonoids, triterpenoids, and tannins in promoting the wound healing by multiple mechanisms, for example, wound contraction, increased rate of epithelialization, and prevention of secondary bacterial infection that would have complicated and delayed wound healing [24, 25]. In the present study, wound healing potency of EECN may be attributed to its high phenolics and flavonoidal content owing to their astringent, anti-inflammatory, and antimicrobial activity. Povidone iodine as standard treatment is a well reported agent as antimicrobial and is used to prevent secondary wound infections. In contrast to that, the EECN extract ointment as already mentioned reveals rich phenolic and flavonoids presence that might have multiple mechanism in favor of wound healing. Collagen is a key extracellular protein in the granulation tissue of healing wound and is the vital component that ultimately plays an important role in wound strength and integrity of tissue matrix [26]. Wound healing process largely depends on the controlled synthesis and deposition of new collagens and their consequent maturation [27]. As wound contraction in EECN treated ointment shows better venerability of collagen synthesis that might be due to the presence of phenolic compounds [28], however the flavonoids might prevent the secondary wound infections as it possesses antiviral and antibacterial activities [29]. In the present study, we evaluate the level of hydroxyproline as a biochemical marker of collagen turnover. Significantly increased (*P* < 0.001) hydroxyproline levels in the granulation tissue of ointment of extract (2% and 5% w/w) treated rats indicate the elevated level of collagen content leading to swift wound healing and this venerable finding might be due to presence of flavonoid [30]. An increase in the tensile strength of the treated wounds was observed and this may be owing to the increased collagen level and stabilization of the collagen fibers [31]. Histopathological study of the ointments treated rat wound tissues also revealed the effectiveness of EECN in improved wound healing.

## 5. Conclusion

In conclusion, the results of the present study revealed that the ethanolic extract ointment of EECN contains the phytoconstituents that promote natural healing process and it could be effectively used as a wound healing agent. EECN ointment efficiently stimulates the wound strength and increases the rate of epithelialization, tensile strength, and collagen viability around the wound area. Further studies are in-process to isolate the active compound(s) responsible for wound healing and efforts shall be taken to develop the commercial preparation for wound healing.

## Figures and Tables

**Table 1:** Percentage yield, phytochemical screening, and quantitative phytochemical standardization of EECN.

Percentage yield	11.78% (w/w)
Phytochemical screening	
Alkaloids	+
Flavonoids	+
Tannins	+
Glycosides	+
Triterpenoids	+
Carbohydrates	+
Steroids	−
Saponins	−

Standardization of content of phytochemicals	
Total polyphenolic content^a^	238.64 ± 1.29
Total flavonoid content^b^	61.39 ± 0.57

For phytochemical screening: (+) presence of phytoconstituents and (−) absence of phytoconstituents; values of standardization of the content of the phytochemicals represent mean ± SD (*n* = 3). ^a^Expressed as mg of gallic acid equivalents (GAE)/g of the dry extract. ^b^Expressed as mg of quercetin equivalents (QE)/g of the dry extract.

**Table 2:** Effect of EECN on % wound contraction and epithelialization period of wound in excision wound model.

Group	% wound contraction				Epithelialization period (days)
	4th day	8th day	12th day	16th day	
Group I (untreated)	4.39 ± 0.82	18.62 ± 0.69	42.62 ± 2.56	68.87 ± 0.91	19.16 ± 0.7
Group II (ointment base treated)	4.21 ± 0.19	19.48 ± 0.93	42.76 ± 1.36	71.2 ± 0.93	19.66 ± 0.66
Group III (standard)	14.55 ± 0.87 a^*∗∗∗*^, b^*∗∗∗*^	38.39 ± 0.46 a^*∗∗∗*^, b^*∗∗∗*^, c^*∗∗∗*^	67.38 ± 2.01 a^*∗∗∗*^, b^*∗∗∗*^	94.3 ± 0.43 a^*∗∗∗*^, b^*∗∗∗*^	17.33 ± 0.4 a^*∗∗*^, b^*∗∗*^
Group IV (2% w/w, ointment)	13.83 ± 1.0 a^*∗∗∗*^, b^*∗∗∗*^	31.11 ± 0.83 a^*∗∗∗*^, b^*∗∗∗*^, c^*∗∗*^	56.46 ± 0.79 a^*∗∗∗*^, b^*∗∗∗*^, c^*∗∗*^	80.21 ± 0.27 a^*∗∗∗*^, b^*∗∗∗*^, c^*∗∗*^	18.33 ± 0.22
Group V (5% w/w, ointment)	14.33 ± 0.94 a^*∗∗∗*^, b^*∗∗∗*^	34.82 ± 0.67 a^*∗∗∗*^, b^*∗∗∗*^	59.64 ± 1.18 a^*∗∗∗*^, b^*∗∗∗*^, c^*∗∗*^	93.58 ± 0.76 a^*∗∗∗*^, b^*∗∗∗*^	17.66 ± 0.7 a^*∗∗*^, b^*∗∗*^

All values are represented as mean ± SEM, *n* = 6 animals in each group. Data were analyzed by one-way ANOVA, followed by Tukey-Kramer Multiple Comparisons Test. a: significant difference as compared to untreated group (group I); b: significant difference as compared to ointment base treated group (group II); c: significant difference as compared to standard group (group III), and ^*∗∗*^ *P* < 0.01, ^*∗∗∗*^ *P* < 0.001.

**Table 3:** Effect of EECN on tissue hydroxyproline content in excision wound model.

Group	Dry weight of tissues (mg)	Hydroxyproline content (*µ*g/100 mg tissues)
Group I (untreated)	42.66 ± 1.13	27.50 ± 0.71
Group II (ointment base treated)	44.31 ± 0.8	28.13 ± 0.92
Group II (standard)	44.33 ± 2.0	34.50 ± 0.84 a^*∗∗∗*^, b^*∗∗∗*^
Group III (2% w/w, ointment)	41.66 ± 1.22	31.83 ± 0.74 a^*∗∗*^, b^*∗∗*^
Group IV (5% w/w, ointment)	39.0 ± 3.12	32.16 ± 0.65 a^*∗∗*^, b^*∗∗*^

All values are represented as mean ± SEM, *n* = 6 animals in each group. Data were analyzed by one-way ANOVA, followed by Tukey-Kramer Multiple Comparisons Test. a: significant difference as compared to untreated group (group I); b: significant difference as compared to ointment base treated group (group II), and ^*∗∗*^ *P *< 0.01, ^*∗∗∗*^ *P* < 0.001.

**Table 4:** Effect of EECN on tensile strength of wound in incision wound model.

Groups	Wound breaking strength (g)
Group I (untreated)	168.35 ± 3.53
Group II (ointment base treated)	172.95 ± 1.24
Group III (standard)	210.76 ± 6.65 a^*∗∗∗*^, b^*∗∗*^
Group IV (2% w/w, ointment)	191.35 ± 6.43 a^*∗∗*^, b^*∗∗*^, c^*∗*^
Group V (5% w/w, ointment)	201.83 ± 4.98 a^*∗∗∗*^, b^*∗∗∗*^

All values are represented as mean ± SEM, *n* = 6 animals in each group. Data were analyzed by one-way ANOVA, followed by Tukey-Kramer Multiple Comparisons Test. a: significant difference as compared to untreated group (group I); b: significant difference as compared to ointment base treated group (group II); c: significant difference as compared to standard group (group III), and ^*∗*^ *P* < 0.05, ^*∗∗*^ *P* < 0.01, and ^*∗∗∗*^ *P* < 0.001.
